# Exploring the need for a new UK occupational therapy intervention for people with dementia and family carers: Community Occupational Therapy in Dementia (COTiD). A focus group study

**DOI:** 10.1080/13607863.2015.1037243

**Published:** 2015-05-01

**Authors:** Sinéad M Hynes, Becky Field, Ritchard Ledgerd, Thomas Swinson, Jennifer Wenborn, Laura di Bona, Esme Moniz-Cook, Fiona Poland, Martin Orrell

**Affiliations:** ^a^ Occupational Science and Occupational Therapy Department, Faculty of Medicine, University of British Columbia, Vancouver, Canada; ^b^ Rehabilitation and Assistive Technology Group, Health Services Research, School of Health and Related Research (ScHARR), The University of Sheffield, Sheffield, United Kingdom; ^c^ Dementia Care Research Centre, Research & Development Department, North East London NHS Foundation Trust, Essex, United Kingdom; ^d^ Centre of Dementia Research and Practice, University of Hull, Hull, United Kingdom; ^e^ School of Health Sciences, University of East Anglia, Norwich, United Kingdom; ^f^ Department of Mental Health Sciences, University College London, London, United Kingdom

**Keywords:** Alzheimer's disease, quality of life/well-being, caregiving and interventions, psychosocial interventions

## Abstract

**Objectives:** In the Netherlands, Graff et al. found Community Occupational Therapy in Dementia (COTiD) demonstrated benefits to people with dementia and family carers. In this study, focus groups took place with people with dementia and family carers to explore how to make COTiD relevant to the UK context.

**Method:** Six focus groups (three with people living with dementia (*n* = 18) and three with family carers (*n* = 21)) took place. Participants were asked for their impressions of the intervention, the extent to which it could meet their needs, and what modifications were needed. Audio-recordings of the groups were transcribed and analysed.

**Results:** Three key themes emerged covering ‘loss and living with dementia’, ‘what helped us’, and ‘consistency and continuity’. People with dementia and family carers spoke about the impact of their diagnosis on them and their family and what strategies helped. Issues such as timing, follow-up, and the importance of an early intervention in preventing crises were highlighted. There was some concern over the length of the intervention and the disruption it might cause to current schedules.

**Conclusion:** Overall, participants were optimistic about COTiD being used in the United Kingdom if it was to be introduced in a flexible and timely manner, incorporating the needs and existing strategies of the person with dementia. These outcomes have led to changes, such as incorporating more flexibility into COTiD, being made to the intervention prior to its implementation in the United Kingdom.

## Introduction

The G8 Summit on Dementia in 2013 prioritised an early intervention and care in the community and people's homes. The UK government has pledged to provide community-based programmes which aim to improve quality of life for people with dementia and their carers (www.gov.uk/government/publications/g8-dementia-summit-agreements, 2013). Training and supporting carers and tailoring interventions to each individual are seen as key to this. Personalised interventions can improve family carers’ well-being, delay admission to care homes, and reduce the risk of institutionalisation by one-third (Olazarán et al., 2010; Spijker et al., 2008). The National Institute for Health and Care Excellence (NICE)/Social Care Institute for Excellence (SCIE) practice guideline for supporting people with dementia and their carers (NICE/SCIE, 2006) recommends advice and skills training from an occupational therapist to help maintain the independence of people living with dementia.

In the Netherlands, Graff et al. (2006) compared the provision of a Community Occupational Therapy in Dementia (COTiD) programme versus a no-occupational therapy group. They demonstrated benefits to activities of daily living (ADL) skills, the quality of life, and depression in people with dementia; an improved quality of life, and enhanced mood and sense of competence in carers; and cost effectiveness (Graff et al., 2008). The COTiD programme (Graff et al, 2010) comprises 10 one hour sessions of home-based occupational therapy provided over five weeks, working in partnership with the person who has dementia and their family carer to improve skills in meaningful daily activities, and caregivers' abilities and sense of competence. COTiD appears to have great potential for adoption in the UK as it addresses key objectives of the National Dementia Strategy. A subsequent study in Germany (Voigt-Radloff et al., 2011) directly translated the Dutch model to German and did not carry out any feasibility or adaption work. They found no difference between providing COTiD or a single consultation, highlighting the need to adapt complex interventions for cross-national comparison and evaluation to be effective. Hence, the need to translate and adapt the COTiD intervention and training programme to maximise its suitability and usefulness within the UK setting before proceeding to a pilot and then a randomised controlled trial. This study forms part of the translation and adaption process and the aim was to employ focus groups with people with dementia and family carers to explore how the COTiD intervention may work with the UK services context and what may need to be adapted to make it relevant to the United Kingdom.

## Method

### Design

Focus groups explore peoples’ views on topics in which they have a vested interest and is increasingly used to develop health care interventions (Kielhofner, 2006) and generate interaction between participants within a group to produce rich data that might not otherwise be collected from individual interviews (Bowling, 2009). Focus group research depends on the interaction within the group for generating data (Kitzinger, 2000). Although the questions are provided by the researchers for discussion, the direction of the discussion and the priority given to the topic can to some extent be controlled by the research participants. The aims of this focus group were as follows:

1. To elicit views of the proposed COTiD programme from people with dementia and family carers and the extent to which the programme may be able to meet their needs and preferences.
2. To identify any aspects that may require changes to make the COTiD programme suitable for use in the United Kingdom.

### Preparation

A topic guide was devised for the focus groups in collaboration with researchers from a variety of backgrounds: psychology, sociology, occupational therapy, and psychiatry. The topic guide was revised several times before the final version was agreed. The main revisions related to length. The final version of the topic guide was shown to members of the Patient and Public Involvement (PPI) group and members of an expert occupational therapy group. The topic guide was designed to explore participants’ views on the content of COTiD; how COTiD is delivered; and potential barriers and facilitators to delivering COTiD in the UK

As participants had no prior knowledge of COTiD and many of them had never had contact with occupational therapy before, short video clips were created to be used during the focus groups to describe COTiD. The videos were between two and four minutes in length and involved a COTiD-trained occupational therapist working with an older couple on different aspects of the COTiD intervention. The couple in the video were actors who had knowledge of the COTiD intervention.

Prior to the first focus group the materials (topic guide and video clips) were shown separately to two members of the PPI group (both former carers) to obtain feedback. Following each focus group the facilitators reflected on the dynamics and issues generated by discussion and reviewed if any changes needed to be made for subsequent groups.

### Participants

To maximise the diversity of different contexts and services, people with dementia and family carers were recruited at three research sites and a sampling matrix was devised to support purposive sampling. Research staff collaborated with relevant NHS and voluntary organisations to promote the study and recruit participants. Participants were recruited through email, telephone, and face-to-face presentations at carer support groups.

To be eligible to participate people with dementia had to be living in the community in their own home (included living with a relative or in a sheltered accommodation) and had an identified family carer who provided at least two hours support per week. Family carers were the primary persons responsible for, and providing practical support (domestic and/or personal) to, a person with dementia, for at least two hours per week, or had done so within the last two years. Both people with dementia and family carers needed to be able to converse in English and had the capacity to provide consent, as well as being able to participate in a group discussion.

Each site varied in the numbers that participated, as can be seen in Table 1. Group one had the largest number of participants. They had an established carer support group willing to participate in research which made recruitment easier than at the other two sites. Participants in all groups were provided with lunch and a small gift (a store voucher) for participating.

**Table 1.** Recruited participants.

	Group 1		Group 2		Group 3	
	PwD*	FC*	PwD	FC	PwD	FC
Number in group	9	13	7	5	2	3
Male	6	4	6	0	1	3
Female	3	9	1	5	1	0
Relationship to PwD – spouse	n/a	11	n/a	3	n/a	1
Relationship to PwD – child	n/a	2	n/a	2	n/a	2

*Note: PwD = person with dementia; FC = family carer.

### Procedures

Three focus groups for people with dementia and three for family caregivers were held. Accessible community venues with a quiet and comfortable seating area were used, in most cases somewhere that participants were already familiar with. Refreshments were provided on arrival. Before the focus group began, informed consent was gained from participants, who were provided with a copy of their signed consent form. The focus groups lasted between 50 and 70 minutes each. Two research staff conducted the sessions: a facilitator led the discussion to try to ensure all participants had opportunities to express their views and a scribe took field notes, recorded non-verbal interactions, and other relevant details during and immediately after the group. Each group was audio-recorded.

Each group began with the facilitator reiterating the purpose of the focus group and discussion of the ground rules. Some informal discussion was then encouraged around ageing and what people find more difficult as they get older. This was then followed by a verbal and video explanation of COTiD -- aims, content, and delivery. Links were made between COTiD and the earlier discussion on ageing. There was discussion around examples of goals that might be identified during COTiD, such as learning to use a mobile phone or cooking a meal with a partner. Participants were then asked to comment on COTiD and also asked what they thought needed to be changed. At the end of the focus group, the facilitator summarised the main points, participants were thanked, and an explanation was given about how the data was going to be used.

### Data

Data consisted of audio-recordings of focus groups, field notes, and staff reflective diaries. Analysis took place at two different sites by two researchers. A timeline was set in collaboration in order to ensure timely analysis of material. Digital recordings were transcribed through an external professional service. These transcripts were checked by researchers who had attended the groups for content, accuracy, and any missing data.

An inductive, data-driven, approach was taken to analysis. Thematic analysis was carried out through rigorous reading and re-reading of the transcripts. From this, a list of codes was generated for each transcript. During the systematic coding of transcripts, data was collated relating to each code. Each researcher listed and explained the codes and themes identified. Key themes were then generated from the codes and revised iteratively by checking their contextualisation within transcripts. Themes were changed and renamed until both researchers were in agreement. They were then defined by the researchers and were iteratively reviewed, dropped, or changed through this process. Following individual unit analysis, cross-case analysis was conducted across the whole data-set. Quotes from transcripts, which related to each theme, were then identified and evaluated for their relevance in evidencing the scope of each theme. Each definition was populated with relevant quotes by researchers to ensure that there were no redundant themes.

## Results

Three key themes emerged: loss and living with dementia; what helped us; and consistency and continuity. These are presented in Table 2 alongside their definitions in this context, linked to the identified codes, and then presented in the remainder of this section illustrated by quotes and analytic commentary. Positive, negative, and ambivalent responses to COTiD were embedded throughout the three themes.

**Table 2.** Themes and definitions from cross-site analysis.

Themes	Definition
Loss and living with dementia	Wide-ranging impacts of dementia and ageing, including the following: • Negative impact of dementia • Social, physical, cognitive, emotional/psychological • Reduced independence with daily activities • Reduced ability to carry out leisure activities/hobbies
What helped us	• What people wanted around the time of diagnosis • What did not help them at the time of diagnosis • Social and leisure activities that people enjoyed/valued • Out-of-house activities • Meeting people in similar circumstances • Respite • Coping strategies • Family support/relationships • Help and support in general, including research
Consistency and continuity	• Need for follow-up • The need for the same occupational therapist to be present throughout COTiD • Different behaviours with different people • May do more/try more with OT than family carer

### Loss and living with dementia

When asked to discuss dementia and living with dementia, all participants spoke about it in negative terms. Participants spoke about how the diagnosis had an impact on both themselves and also their family. This area of discussion appeared to matter most to them and some participants were especially emotional when speaking about their experiences of living with dementia, as seen in this man's words:

You feel very stupid sometimes when it's. You'll say something and then you completely forget what you were going to say…I can't get the words out, you know I'm stuttering now, I don't know what to say.

Difficulties with memory, concentration, and attention were reported in all groups and as a particular source of frustration for people. Detailed examples of increasing impairment were given of how people with dementia had to rely more on others because of memory problems, stop reading novels because of difficulties with concentration, and finding more difficulty in following conversations because of problems with directing their attention. For many, these cognitive problems were the first difficulties that they experienced and so would have been present for a long time. No one stated that this was something that they got used to but did describe ways in which they did try to adapt and compensate for their difficulties.

Participants spoke about trying to get used to not being able to carry out previous roles such as being a cook of the house or DIY expert. These roles had to be given to others which was a difficult transition, as below:

I was in the building trade all my life and recently we've had to get people to come and do the jobs at our house now…It sort of hits you like, oh you sort of realise that you can't do what you want. I've been doing it for thirty years, why can't I still do it now?

These comments emphasise their sense of breaking with and loss from their own sense of their life history up to then. As well as key household roles, participants spoke about having to stop previously enjoyed hobbies due to cognitive issues as well as physical difficulties relating to ageing. They expressed a genuine sense of loss where previous activities had stopped:

Managing other people, I can't do that anymore…some say I can't manage myself…organisational way. I used to be involved in stuff at church but I'm no longer reliable, I can't do it anymore.

This woman describes herself as having lost the valued characteristic of ‘being reliable’, an increasing negative aspect of living with dementia. Despite the negative views of dementia held by all group members, there was still a strong resilience expressed by participants in their ability to adapt in order to cope and continue living with dementia, as one asserted: ‘I feel I've still got a lot of life left in me.’ Within the groups, tips and services that were useful were shared by fellow participants and people expressed their appreciation for this. There were also people who wanted the space to air their grievances -- in particular, at the health services.

There was some concern expressed by older family carers, who themselves might have health problems, over their ability to care for their husband/wife in the future. They worried that if anything happened to them they would not be able to carry on in their caring role and this could separate the couple. For a number of participants in the group, this was something that was reported to be at the forefront of their minds constantly:

Your physical capabilities and maybe what you want to do are not going to always match up.

Similar concerns were expressed by the younger family carers this time in terms of how their father or mother would cope if they became ill. Because of the demands that are placed on people as carers, it was not something that people thought they could continue with if their health got worse. For many participants, the relationship they had with their family changed with dementia, but this was not necessarily always experienced as a negative change. One participant was adamant that his relationship with his wife had not changed and would not change as a result of the diagnosis of dementia:

The relationship between my wife is just the same it has not changed, except that her memory is not working properly. So I still love my wife, I cherish her. I do love her, I do tell her and all that, so that relationship is still there, it has not gone. It is always there and will continue to be there until maybe the time that she won't remember me, but I don't know when that is going to be, it might never be.

### What helped us

People with dementia and family carers reported different ways of dealing with the changes that dementia had brought into their lives. As participants in the focus groups had dementia for varying lengths and their situations and relationships differed, a contrast could be seen in the strategies employed by participants. For one family carer, and his wife, receiving the diagnosis was in itself helpful for them:

When he came here and was diagnosed he said ‘well thank goodness I know what's happening now, I thought I was going mad.

People with dementia and family carers expressed their preference for more support to be provided post-diagnosis and signposting to relevant services. There was some frustration over being given a diagnosis and then left by services for months at a time to digest the information and cope alone. Some services were found easier to access than others and some participants reported being more proactive than others in seeking our support. However, one participant actively avoided seeking help and support and expressed his preference to care for his wife alone as he felt best placed to do this.

Support from family was seen as key to coping, and was described as being what helped people most both emotionally and physically. People with dementia increasingly relied on their spouses and rarely left the house alone since receiving their diagnosis and so groups raised their concerns for participants living alone. Those participants who themselves lived alone, however, while admitting that they struggled at times, still did not want to burden anyone with their difficulties, unless it was unavoidable:

I don't want to be a bore to the children and say oh what's this and what's that and where do I put this and do that. I want to try and do it myself. It might take a little bit of time, whatever it is, but I'll do it, I'm an independent type of person and I prefer that.

This participant underlined the value that they placed on maintaining their independence. Participants who lived alone described the strategies they employed to cope which included talking to themselves to keep in mind what they were doing and using calendars and diaries. They also highlighted that they made an effort to ensure that they were involved in activities -- both new and old outside their home. People with dementia reported taking up new hobbies and becoming involved in various social clubs and activities that they had not previously done such as a men's club and a walking group. These people found the groups to be a good form of support, healthy activity, and socialising. People with dementia appreciated meeting people in similar circumstances to themselves. Many had not had experience of dementia before and said they had learned a lot from meeting others.

I was just going to say that one of the things that's helped me a lot is being around people that are fighting dementia problems and the self-help groups are very useful in that. I'm also a volunteer at ……I find it very encouraging to see people fighting and coping with it and that's a useful end in itself.

People with dementia spoke about COTiD potentially benefiting them by supporting them to retain previous hobbies and to take up new hobbies. People were also keen to do something to help themselves remain independent. Some participants were ambivalent about COTiD as they found it difficult to apply the programme to their current needs. One person with dementia here stated that he could not see how it would help him with his everyday difficulties like remembering where the salt was kept:

I can't understand how they could get to my mind to put the salt in there instead of putting it over there.

Participants welcomed the idea that partnership between the occupational therapists and the person with dementia and family carer would be enabled by the proposed intervention. This seemed to be important to people with dementia in terms of maintaining their autonomy and decision-making and for the family carer because their difficulties were being acknowledged and addressed, as stated by this family carer:

I think the good thing … is looking at the couple together… carers become so stressed by the time it comes to having to sort things out, that you've actually sometimes lost that power to think logically yourself…if you had someone there guiding you through that and helping you with that I think that is a massive thing to help and recognise that it is a stress to the carer as well….

There was an importance placed on supporting and guiding the carer throughout COTiD. Their preceding experiences of health services have routinely placed the focus of health care interventions solely on the person with dementia so that the family carer's concomitant needs for support could sometimes be forgotten. This person with dementia explains why they think it is important to include the family carer:

But the other person, the person that's caring for you, they might get some help out of it as well, and I think that's a significant thing so, it's the carer that might find ways forward and help them cope with your dementia.

This participant highlighted the encouragement they gained from seeing others countering and coping with problems experienced in living with dementia. Taking part in the focus groups and other research projects was in turn also identified as something that people found helpful. They not only enjoyed the social aspects of the group, but valued the sense that they were contributing to dementia research. Many expressed the view that while the research may not benefit them directly they wanted to be a part of the research process to help others who may be in their situation in the future.

This is for research and obviously it might not do us any good, but it might do future people good.

### Consistency and continuity

Based on the previous experience of services, two related issues featured in all group discussions: the need for continuity of support throughout the dementia pathway and also for consistency of approach. Many people found the services that they dealt with fragmentary and inconsistent in the support provided and that they had been left to deal with the diagnosis after seeing the specialist doctor. In dealing with health services to date, participants felt that they were constantly asked the same questions and they thought that there was little or no communication between professionals. Having the same therapist throughout the 10-week COTiD intervention would go some way to having some consistency of approach:

That is what they need, continuity. Different people coming in wouldn't work.

Both family careers and people with dementia emphasised the importance to them of follow-up to see how people were managing in their daily lives. For COTiD they suggested having a number of follow-up sessions at varying intervals after the last intervention session to ensure that people are implementing what has been worked on and to see if further support is needed.

The time commitment involved in COTiD was an area of contention for some participants in each group. There was a common opinion expressed that there would be too many and that there would be difficulty fitting something new into an already full weekly schedule as stated by a family carer here:

It's trying to find an hour free. When you have got somebody who has also got other illnesses apart from dementia, next week I haven't got a day free.

Family carers saw the importance of having the same therapist involved in each stage of the process. They said that the therapist may be able to get people to do things that they can't get them to do and the value that a different approach might bring to a difficult issue:

It might be that if the therapist came to our house she could get my wife more motivated than I can. Because it's a stranger saying do this, do that.

Family carers stressed the importance of having the person with dementia motivated in order for the programme to be successful. There was some concern that without this there would be no benefits seen, as discussed between two family carers here:

I think it's the willingness of the person with dementia to take part really…I think if you can achieve that then we might make some strides.

I couldn't agree any more than that, I think that sums it up perfectly.

## Discussion

This study found that participants wanted COTiD to be flexible, consistent, fit within their existing demands, and include the person and their carer as partners when delivering interventions. The results here suggest that to date participants have, on a whole, been unsatisfied with the support they have received from health services. The importance placed on an early intervention by both people with dementia and family carers and that many people reported not having received adequate support and signposting following diagnosis of dementia are important for health care providers to be aware of. In order for any therapeutic process to be successful, support needs to be provided through the dementia pathway to both the person with dementia and the family carer.

Three main themes emerged from the focus groups: loss and living with dementia; what helped us; and consistency and continuity. Participants in both groups expressed their appreciation of having space and time to speak about their experiences of dementia within the groups. Their views of living with dementia were largely negative, which is consistent with previous studies (von Kutzleben et al., 2012), but they also articulated many ways in which they had adapted constructively to their current situation. In dementia people experience multiple losses (Basting, 2003; von Kutzleben et al., 2012) as well as increasing their need for help from others (Cotrell & Hooker, 2005; MacQuarrie, 2005). Although some people became upset when discussing the impact that dementia had had on their lives, in many ways they were managing well. A person's self-esteem is often affected when they lose some of their skills and become less competent at tasks (Fazio & Mitchell, 2009) and this can often be upsetting for people to discuss. COTiD aims to help people work on these skills which may impact on quality of life, as well as self-esteem.

In these focus groups, family carers viewed the intervention as being able to enhance their skill set and equip them better to face challenges that they may encounter in the future. Van Gennip and colleagues (2014) found that in order to maintain the autonomy that they have, people with dementia need to rely more and more on friends, family, and health services. This indicates the importance of including the family carer in the intervention when the goal is to maintain the independence of the person with dementia, which is a key factor in COTiD. The issue of supporting the family carer along the dementia pathway was something that emerged from the focus groups with both people with dementia and family carers. The involvement of the family carer in the intervention has the potential to increase their sense of competence (Graff et al., 2008), and research to date has shown that carers are a group who are under a huge amount of stress as a result of the impact of caring (Ferri et al., 2005). The person with dementia's level of functional impairment and behavioural issues impacts on the carer's level of stress (Donaldson, Tarrier,& Burns, 1997; Kneebone & Martin, 2003) and so working with the family carer to help manage limitations and reduce behaviours that challenge should benefit both the person with dementia and the family carer. Furthermore, a systematic review of the influence of relationship factors on people with dementia and their family carers by Ablitt, Jones,and Muers (2009) found that joint interventions had the added bonus of automatically including relationship factors through working towards common goals and support the person with dementia and family carer in working together.

Most of participants’ suggestions for further refining COTiD were based on their experiences of health and social care services. Perhaps unsurprisingly, the suggestions for change were consistent across people with dementia and family carer groups. Issues such as timing, follow-up, and consistency of approach were identified as important to participants. Joint interventions have the potential to have a flexible approach to timing and the number of sessions, as well as aiming the intervention at the specific needs of the person with dementia and the family carer (Ablitt et al., 2009).

Steeman and colleagues (2006) found that consistent care and follow-up services were essential to live well with dementia. They suggested that care should be proactive and involve people close to the person with dementia so that they have someone to go through the adjustment process with. Gill, White, and Cameron (2011) interviewed 22 people with dementia to gain their perceptions of the interactions that they have had with health care. They found that people were keen to express their views on the topic and that services need to work flexibly with the client so that the intervention is a good fit for everyone. This idea of fitting the intervention to the person is core to COTiD and Gill et al. (2011) also state that health care practitioners should be trained in ways to ensure such tailoring occurs.

An important finding was participants’ willingness to be involved in research. Involving people with dementia in research is relatively new (Nygård, 2006), where previous research relied on proxy views. Participants felt it was important that they were trying to make a difference to the lives of other people with dementia. Participants take the risk, through the informed consent process, of speaking about potentially distressing matters (Woods & Pratt, 2005) during the research process. Their motivation to participate appeared to be altruistic as many participants said explicitly that they did not think that current research would benefit them but that they wanted to help make things better for people with dementia in the future. A systematic review of the subjective experiences of people with dementia living in the community (von Kutzleben et al., 2012) showed the recent shift in scientific processes to include the views of people with dementia in research (e.g.,Aggarwal et al., 2003) and the significance of the subjective experience to both qualitative and quantitative work. In order to develop any needs-based interventions, it is imperative that the group receiving the care be involved in consultations. This was the purpose of carrying out the focus groups and the research team aim to continue this with other aspects of the COTiD project.

### Limitations and methodological challenges

A number of challenges were encountered through using a focus group design. Success in recruiting adequate numbers of participants varied greatly according to the type and location of groups. While some groups had more participants than anticipated, some had too few. The challenges of having a bigger group were in ensuring that everyone's views were included. The facilitator managed this by encouraging participants to allow different people to speak, keeping answers brief when possible, and directly asking the quieter members if they wanted to add to the discussion. There were some challenges in keeping the bigger group on-topic and needed to be directed back to topics or questions more often as discussions proceeded. The discussions that took place within the smaller groups did still benefit from having a group format as people discussed the issues together and there was a sense that the smaller group made it easier for people to have their views heard. The population sampled was also not a diverse one. All except one participant described themselves as ‘White British’ and the person with dementia was cared for by either their spouse or adult child. The different challenges and priorities that came from caring for different groups of people were included as a result.

Participants had no direct knowledge of COTiD which made it challenging for the facilitator to convey the process to participants in a way that everyone understood. Some people with dementia found it difficult to consider what was essentially a hypothetical situation. This was addressed by the researchers by giving a number of different examples and relating the examples to the areas of difficulty that they reported at the beginning of the group. Goldsmith (1996) gives guidance on communicating in research with people with dementia such as listening attentively, accepting the person for who they are, and being open during the process when people are sharing their views. Many of the techniques suggested by Goldsmith (1996), such as introducing people, calling people by their names, and being comfortable with long pauses and displays of emotion, were followed. Despite the challenges, the benefits of including people with dementia in research activities cannot be underestimated – very valuable information that will be key to the future of the COTiD programme was gained by undertaking these focus groups.

## Conclusion

People with dementia and family carers’ views highlighted that a consistent approach, early intervention, and including, when appropriate, people's previous occupations, need to be considered for COTiD to be appropriately adapted and implemented in the United Kingdom. People with dementia and family carers were supportive of implementing COTiD in the UK aspects; however, family carers indicated some areas of concern, such as decision-making for people with dementia. The findings of this study highlight not only the important role of people with dementia and family carers in the development of the COTiD intervention, but also all aspects of dementia care. This includes giving family carers a more active role in intervention, working flexibly with families, maintaining motivation to participate, and finding ways to incorporate their existing coping strategies. Future research should also ensure that people with dementia are included in decision-making about the design and application of new interventions and service. Key suggestions, such as introducing some flexibility around the timing and length of the intervention, and ensuring that people are recruited to COTiD at the appropriate stage in the dementia pathway, have been prioritised and integrated into the final adaptions of COTiD-UK to be used in a randomised controlled trial in order to make it more relevant and useful to people in the United Kingdom.
